# Considerations for study design and analysis for ethically and culturally safe DNA methylation research in Aotearoa New Zealand

**DOI:** 10.1016/j.ssmph.2025.101889

**Published:** 2025-12-04

**Authors:** Anna Rolleston, Gregory T. Jones, Nikki J. Earle, Sam Gibbs, Anna Pilbrow, Allamanda Faatoese, Katrina K. Poppe, Kimiora Henare, Vicky A. Cameron, Donia Macartney-Coxson, Malcolm E. Legget, Robert N. Doughty

**Affiliations:** a The Centre for Health, 103 Third Ave, Tauranga, 3110, New Zealand; b Department of Surgical Sciences, Dunedin Medical School, University of Otago, 201 Great King St, Dunedin, 9016, New Zealand; c Department of Medicine, University of Auckland, Private Bag 92019, Auckland, 1023, New Zealand; d Christchurch Heart Institute, Department of Medicine, University of Otago, Christchurch, PO Box 4345, Christchurch, 8140, New Zealand; e Auckland Cancer Society, Auckland Cancer Society Research Centre the University of Auckland, Auckland New Zealand Research Centre, University of Auckland, Private Bag 92019, Auckland, 1023, New Zealand; f New Zealand Institute of Public Health and Forensic Science (PHFScience, formerly ESR), 34 Kenepuru Drive, Porirua, Wellington, 5381, New Zealand; g Te Toka Tumai Auckland Hospital, 2 Park Rd, Auckland, 1023, New Zealand

**Keywords:** Epigenetics, DNA methylation (DNAm), Cultural safety, Indigenous health research, Ethical frameworks, Data sovereignty

## Abstract

Epigenetic research, particularly DNA methylation (DNAm), holds significant potential for improving cardiovascular disease (CVD) risk prediction, yet its application must be guided by ethical and culturally responsive considerations. This paper examines the integration of a values-based framework to ensure the culturally safe conduct of DNAm research within the Multi-Ethnic New Zealand Study of Acute Coronary Syndromes (MENZACS) cohort. Grounded in Te Tiriti o Waitangi principles and kaupapa Māori methodologies, this study emphasises equity, social accountability, and indigenous data sovereignty. This study was not designed as a discovery epigenome wide analysis, but rather performed, as an exemplar, a SWOT analysis that identified both the potential of DNAm markers, such as cg05575921 in AHRR for smoking exposure assessment, and key risks, including genetic confounding, population-specific variation, and the potential for individual and transgenerational stigma. Findings underscore the importance of ensuring multi-ethnic validation of DNAm markers to prevent exacerbation of health inequities. This paper advocates for the adoption of ethical, culturally attuned research frameworks in epigenetics to enhance equitable health outcomes and support Māori health advancement.

## Highlights

• DNA methylation shows potential to enhance cardiovascular disease risk prediction.
• Novel values-based framework developed to guide culturally safe DNAm research.
• Indigenous principles ensures culturally attuned interpretation of epigenetic data.
• Multi-ethnic validation of DNAm markers is essential to prevent health inequities.
• The cg05575921 AHRR marker is a robust, equitable way to assess smoking exposure.

## 1 Introduction

Epigenetics, the study of changes in gene function that do not involve alterations to the DNA sequence, is an emerging area of research in understanding complex diseases like CVD (Muka et al., 2016). Epigenetic mechanisms, such as DNAm, histone modifications, and non-coding RNA regulation, are key contributors to the development and progression of CVD (Ordovas & Smith, 2010). These changes are influenced by environmental exposures and lifestyle factors, offering insights into disease mechanisms. Understanding these epigenetic processes presents opportunities for early prevention, personalised medicine, and new therapeutic strategies for CVD (Schiano et al., 2020). Changes in DNAm have drawn particular attention in this regard due to robust associations with conditions such as diabetes, dyslipidaemia, metabolic syndrome, rheumatoid arthritis, Alzheimer's disease and breast cancer (Braun et al., 2017; Juvinao-Quintero et al., 2021; Wei et al., 2021). As a developing field in health research, epigenetics therefore appears to hold great potential for improving risk assessment, screening, and resultant health outcomes in cardiovascular care.

However, the application of epigenetic research is not without its challenges, particularly with regard to ethical and social implications. One of the key concerns is the potential for findings to contribute to the stigmatisation of certain population groups. This risk is heightened in research involving already stigmatised or discriminated groups, or when the research questions have normative implications (Hemsley, 2020). Consequently, epigenetic data could inadvertently reinforce harmful narratives, placing blame for health conditions on individuals due to lifestyle factors, such as smoking, while ignoring broader social determinants of health (Evans et al., 2021). Epigenetic findings can also have the potential to direct blame for an individual's risk of chronic disease on their parents, especially mothers (such as associations with maternal smoking) (Everson et al., 2021; Rauschert et al., 2019). This raises important ethical questions around how findings are interpreted and applied in public health and clinical settings.

Furthermore, it is crucial that the benefits of DNAm and other epigenetic discoveries are equitably applicable across diverse populations. In this paper we utilised epigenome wide association study (EWAS) data to demonstrate how a culturally attuned research framework could govern design, analysis, and interpretation of such investigations. It is important to note that this is not intended as a report of a discovery analysis, but rather as a discourse regarding the principles for the adoption of a culturally attuned research framework. Throughout this paper, we use the term *ethnicity* to refer to self-identified population groupings that share ancestry and/or social and historical experiences, and *culture* to refer more broadly to the values, beliefs, knowledge systems, and practices associated with these groupings. Historically, genetic and epigenetic research has often overlooked ethnic and cultural differences, leading to findings that may not be applicable to all communities. Epigenetic changes can be both dynamic and reversible, making them suitable for therapeutic targeting. However, markers identified in one population may not have the same predictive or therapeutic value in others (Kelly et al., 2010; McGuinness et al., 2012) due to under-pinning genetic, or other, variation (Maunakea et al., 2024). This underscores the need for inclusive data sets that reflect the full diversity of human population, ensuring that health advancements are relevant and beneficial to all. This is of particular importance in Aotearoa New Zealand where indigenous Māori experience significant health disparity and where there is a growing multi-ethnic population that includes peoples from the Pacific Islands and Asia.

The Multi-Ethnic New Zealand study of Acute Coronary Syndromes (MENZACS) (Earle et al., 2023) is a prospective longitudinal cohort study of patients presenting with first time acute coronary syndrome (ACS) to New Zealand hospitals between 2015 and 2024. The aim of MENZACS is to determine how genomic, epigenetic and environmental CVD risk profiles differ according to ethnicity, age and sex, and to test their utility for inclusion in clinical models for prediction of secondary events. While any patient presenting with first-time ACS was eligible for inclusion in the study, recruitment strategies were augmented to increase the proportion of Māori and Pacific participants. The study leadership included a Māori Governance Group (MGG) established at study inception.

This paper describes the process of developing ethical and culturally grounded values to guide epigenetic research; we use our DNAm work with the MENZACS cohort in Aotearoa New Zealand as context. C*ultural safety* in this setting originates from critiques of health and education systems and is defined by participants themselves. It emphasises environments where identity is respected and power imbalances addressed. In line with recent work which demonstrates how social inequality and race shape epigenetic aging (Yannatos et al., 2023), we extend equity-aware epigenetic research into an Indigenous, multi-ethnic context. Rather than offering a full empirical paper this work presents exemplars of how these values have informed design, analysis, and reporting of DNAm observations in this multi-ethnic ACS cohort.

## 2 Methodology

A detailed description of the DNA methylation and genotyping laboratory and bioinformatic methods, including quality control and pre-processing pipelines, is provided in the Supplementary Materials. In brief, genomic DNA from whole blood was analysed using Illumina Infinium MethylationEPIC BeadChips (version1) following manufacturer recommendations. Raw idat files were processed using the Illumina GenomeStudio software (default background correction, normalisation, and probe filtering), and the Bioconductor packages ChAMP (Tian et al., 2017) and minifi (v1.46) (Aryee et al., 2014). White blood cell composition (BCC) proportions were estimated from array methylation data using the Houseman extended reference-based method (Houseman et al., 2012; Jaffe & Irizarry, 2014). These estimates, along with chronological age, biological sex, array slide and position, were used as covariates in the EWAS analysis to minimise confounding. Known cross-reactive and non-specific probes (Pidsley et al., 2016a) were excluded from subsequent analysis, however probes associated with known polymorphic CpG sites (Pidsley et al., 2016b) were retained as these were potentially relevant to the subsequent multi-ethnic comparisons. Adjustment for multiple comparisons was made using a false discovery rate (Benjamini–Hochberg) approach and were reported as q-values (significant <0.05).

Genotyping was performed using the Illumina Infinium Global Screening Array (v3.0) (Illumina Inc. San Diego, CA). Imputation was carried out using the Michigan Imputation Server (https://imputationserver.sph.umich.edu/) resulting in over 30 million markers that had an imputation quality (r^2^) of >0.3.

Ethnicity of participants was determined from National Health Index (NHI) records. Recognising the potential for inaccuracies in NHI-recorded ethnicity, we also collected information on grandparent ethnicity at enrolment. Where discrepancies were identified, ethnicity was verified and corrected. Categorising health data by ethnicity is standard practice in Aotearoa New Zealand. The four groups reported here (Māori, Pacific, Indian, and European) represent the population groups with sufficient numbers for analysis and with well-documented differences in cardiovascular outcomes. We acknowledge that the term “Pacific” encompasses multiple distinct nationalities and cultural groups, and use it here as a broad category while recognising the diversity it represents.

To guide the ethical and culturally sensitive conduct of epigenetic research, a MENZACS epigenetics subgroup was convened. This approach aligns with recent findings that emphasize the importance of Indigenous-led, contextually relevant biomedical research that resonates with local health priorities (Wells et al., 2024). The group comprised members from the Māori Governance Group, the MENZACS steering group and epigenetic scientists. Before initiating any research, the group engaged in a series of iterative, consensus-based discussions to explore potential risks, drawing on both the expertise of those present and relevant literature on epigenetics, Indigenous research ethics, and data sovereignty. Through this process, a *mitigation strategy* was developed (referring to a proactive approach to identifying and addressing potential ethical challenges such as stigmatisation, inappropriate interpretation, or inequitable application of findings). The output of this strategy was the co-development of a set of guiding values that would shape all aspects of the research process, from analysis design to interpretation and dissemination. These values underpin a culturally safe, kaupapa Māori (research performed by Māori, with Māori, and for Māori) aligned approach to epigenetic research within a multi-ethnic cohort.

1. **TE TIRITI O WAITANGI** (Partnership) – The research team will ensure decision-making is shared at all stages of the project through open and frequent communication with the MENZACS Māori Governance Group (or their delegated Chair). This may mean frequent meetings at crucial times, such as designing the research questions, guiding direction of the data analyses and interpretation of the findings.
2. **NGĀKAU TAPATAHI** (Integrity) - The research will adhere to all ethics approvals and consents, considerations of confidentiality, and will honour indigenous processes and protocols throughout the project (especially with regards to sample handling), recognising that multiple stakeholders with differing cultural views may be involved.
3. **AROHA KI TE TANGATA** (Social Accountability) – The research team are accountable to the participants and their communities. The core research objectives are to maximise gains in mātauranga Māori, achieve health benefits through a focus on clinically relevant research questions and protect indigenous intellectual property, while identifying and minimising any potential harm to participants and their communities.
4. **MANA** (Dignity/Empowerment) - The research will be considered in the context of colonisation, and the team will be guided by the Māori Governance Group as to how to acknowledge the impact of dispossession of traditional lands, intergenerational trauma, experiences of racism and social deprivation, and reflect a Māori worldview.
5. **TAUUTUUTU** (Reciprocity) - This value requires that the research findings are returned to the communities who contribute to it. In practice, this includes: returning findings in accessible formats, involving Māori leadership in interpretation and dissemination, supporting Māori research workforce development, and upholding Māori data sovereignty.
6. **TE TīMA** (The Team/Capacity Building) – The research team will take every opportunity to strengthen the Māori health research workforce by having Māori researchers involved at all stages of the work, and to advance the career development of existing team members through support by senior colleagues in mentorship, skill sharing and leadership opportunities.

These values provided a framework for conducting, analysing, and reporting epigenetic research, ensuring that all aspects of the study were aligned with a kaupapa Māori research approach. The values were informed by key elements from the CONSIDER statement, a checklist designed to strengthen reporting in health research involving Indigenous peoples (Huria et al., 2019). These values were intended to guide the entire research process, from determining the research questions to analysing the data and interpreting and disseminating the findings. The values also emphasize the importance of inclusivity and cultural sensitivity in epigenetic research, particularly when assessing the relevance of potential markers across diverse populations.

Using this values-based framework, the first step was to conduct a strategic planning technique of assessing strength, weaknesses, opportunities and threats (SWOT) analysis (Nutt & Backoff, 1993) of potential strengths, weaknesses, opportunities and threats regarding the examination of epigenetic (specifically DNAm here) markers in the context of chronic disease risk in Aotearoa New Zealand.

### 2.1 Potential strength & opportunities of epigenetic markers

#### 2.1.1 More precise assessment of disease-related risk factors

Tobacco smoking exposure was identified as a key exemplar for testing DNAm risk markers alongside established clinical, lifestyle and other biomarker risk variables. Current primary (such as PREDICT (Pylypchuk et al., 2018)) or secondary (SCORE2 (SCORE2 working group and ESC Cardiovascular Risk Collaboration, 2021)) cardiovascular risk prediction tools, rely on patients' self-reported declarations of their smoking history, which can be subjective and prone to misclassification (Pell et al., 2008; Wagenknecht et al., 1992). DNA hypomethylation of the CpG site cg05575921 (within *AHRR*), has been shown to be a robust indicator of tobacco smoking exposure (Gao et al., 2015; Fitzgerald et al., 2023), thus DNAm scores may offer objective indicators of tobacco exposure, allowing for more accurate stratification of individuals' exposure and, by extension, a more refined assessment of their cardiovascular risk related to smoking (Lee et al., 2017; Shenker et al., 2013).

In addition to smoking exposure, several inter-related cardiometabolic risk factors, including diabetes, increased adiposity, hypertension, inflammation and dyslipidaemia were also identified for potential analysis due to their known associations with CVD (Guembe et al., 2020). As with smoking assessments, there is recognition that many of these markers could also benefit from refinement.

#### 2.1.2 Accessibility

Most DNAm blood tests are typically performed using whole blood collected via conventional venepuncture (as was the case in the current MENZACS study). However, due to the relative chemical stability of DNAm it has also been shown to be possible to accurately assess DNAm in dried blood spots on cards, even after storage for many years (Cameron et al., 2023). This suggests that testing DNAm markers may be amenable to self-administered finger prick blood sampling, which could in turn increase accessibility by ameliorating the need to attend clinic in person.

### 2.2 Potential weakness and threats of epigenetic markers

#### 2.2.1 Epigenetic markers may not aid risk prediction

Epigenetic markers may not have sufficient independent risk association to substantially improve current risk prediction algorithms. Alternatively, DNAm markers may not change in response to pharmacological or behavioural interventions and therefore not help evaluate risk mitigation.

#### 2.2.2 Potential confounding effect of genetic variation on DNAm

Genetic variants have been shown to be significant potential confounders of observed methylation values (Bhat & Jones, 2023; Villicana & Bell, 2021). These can effectively ablate the presence of the targeted DNAm CpG site. Alternatively, DNAm detection platforms that utilise DNA-binding probes (such as the widely used Illumina Infinium arrays) could be influenced by genetic variants adjacent to the CpG site within the probe recognition region, thereby altering detection efficiency. While both of these situations are recognised as potential confounders, they can be difficult to detect, particularly when the genetic variant has a low allele frequency (Surachman et al., 2024). As such, genetic variation is a substantial potential concern with regards to DNAm markers and their subsequent application as individualised precision-medicine risk tools.

#### 2.2.3 Equitable application of DNAm markers across ethnic groups

If such genetic effects involve variants with population-specific allele frequencies there is a further risk of producing inequitable outcomes, by generating inaccurate risk predictions within specific population groups. This is particularly true if the DNA marker does not have substantial genetic variation in the discovery cohort but is then applied to another population with a different ancestral background that has a confounding genetic variant. Given the limited reference genome data available for Māori and Pacific populations, this was identified as a significant area of concern.

#### 2.2.4 Stigmatisation (individual and transgenerational)

Using DNAm for risk profiling can result in findings that could be stigmatising to an individual or group, and care must be taken in disseminating results to avoid reinforcing harmful narratives. Such issues include:

Maternal environment (such as smoking (Everson et al., 2021; Rauschert et al., 2019), alcohol (Surachman et al., 2024), illicit drug use (Oni-Orisan et al., 2021)).
Personal alcohol consumption (Rosen et al., 2018).
Dietary quality
  Individual's dietary exposure (Garcia-Garcia et al., 2024; He et al., 2019).
  Transgenerational effects of famine exposure (Gonzalez-Rodriguez et al., 2023).
Education attainment and socioeconomic status.
  DNAm markers have been suggested as indicators of socioeconomic (Cerutti et al., 2021; Rajaprakash et al., 2023) and educational attainment, including a potential transgenerational effect (Surachman et al., 2024).

#### 2.2.5 Data sovereignty

Data sharing has emerged as a strong expectation in most biomedical research disciplines (Tedersoo et al., 2021), with data being made publicly available often being a condition of publication. This may be incompatible with principles of data sovereignty for Indigenous Peoples (Hudson et al., 2023; Lilley et al., 2024). The need to upload data to externally hosted servers to calculate some ‘epigenetic clock’ scores may therefore represent a challenge to the utilisation of such tools. Recent advances to make leading epigenetic scores, such as GrimAge2, more openly available to the global research community are therefore to be encouraged.

## 3 Operationalising guiding values

To support transparency and broader adoption of culturally responsive epigenetic research practices, we explicitly mapped the guiding values underpinning this study to the CARE Principles for Indigenous Data Governance (Research Data Alliance International Indigenous Data Sovereignty Interest Group, 2019) and the domains of Te Mana Raraunga (Te Mana Raraunga, 2018). Table 1 illustrates how each value was operationalised in practice, including specific governance mechanisms and protocols applied in the MENZACS study. The mapping serves to demonstrate how ethical intent was embedded throughout the research process, from study design to data management and dissemination.

**Table 1:** Mapping MENZACS epigenetic study values to Te Mana Raraunga and CARE Principles.

Study Value	CARE Principle	Te Mana Raraunga Domain	Practice in MENZACs
**TE TIRITI O WAITANGI** (Partnership)	Collective Benefit, Ethics	Rangatiratanga, Kotahitanga, Whanaungatanga	MGG established at inception; co-design of protocols and framing; governance of subgroup; joint authorship and decision-making (delegated approval pathway).
**NGĀKAU TAPATAHI** (Integrity)	Ethics, Responsibility	Kaitiakitanga, Manaakitanga	Data stored in secure-compute locations. No data uploaded to offshore servers for clock scores; ongoing ethical review; transparent reporting of risk mitigation strategies; ethical literacy across team.
**AROHA KI TE TANGATA** (Social Accountability)	Responsibility, Ethics	Whanaungatanga, Kaitiakitanga	Transparent discussions on risks of stigmatisation; dissemination of findings in culturally appropriate formats; alignment with collective Māori interests.
**MANA** (Dignity/Empowerment)	Collective Benefit, Authority	Manaakitanga, Rangatiratanga	Inclusion of whakapapa and grandparent ethnicity to honour participant identity; support of culturally safe reporting and analysis.
**TAUUTUUTU** (Reciprocity)	Collective Benefit, Ethics	Manaakitanga, Kotahitanga, Kaitiakitanga	Community-facing dissemination of findings; shared interpretation of results with MGG.
**TE TīMA** (The Team/Capacity Building)	Collective Benefit, Responsibility	Kotahitanga, Kaitiakitanga	Māori health research workforce development via mentored co-authorship, analytic capability support, and MGG leadership.

## 4 Findings

### 4.1 Values guided approach to DNAm analysis of the MENZACs cohort

We performed an epigenome-wide analysis examining a subset of 979 MENZACS participants (including 265 Māori, 98 Pacific, 91 Indian and 525 European) using the Illumina EPIC DNA methylation (v1) array.

First, we interrogated the data to examine DNAm associated with (never, ex- or current) smoking status in Europeans in the MENZACS cohort. This analysis significantly replicated previous reports suggesting strong consistent smoking status associations with DNAm CpG sites in gene region such as *AHRR, F2RL3, RARA, PRSS23, IER3, GPR15* (Gao et al., 2015; Pospiech et al., 2024). Also concordant with previous reports, the strongest association was with the DNAm CpG site cg05575921 in *AHRR.* The next step was to evaluate the same epigenome-wide smoking associations within the specific MENZACS population groups, from which cg05575921 *AHRR* was also confirmed as the strongest marker in Māori, Pacific and Indian (Supplementary Fig. 1). An important principle in this analysis was that the initial discovery was made in a muti-ethnic fashion, confirming that the ‘top-hit’ marker discovery was the most relevant for each of the specific population groups, rather than simply validating the European discovery.

Following the generation of these results, we then revisited our values-based framework before progressing to any further validation or translational analysis. This iterative pre-analysis discussion process was applied to each phase of the study.

### 4.2 Genetic effects on DNA methylation

**Fig. 1:**
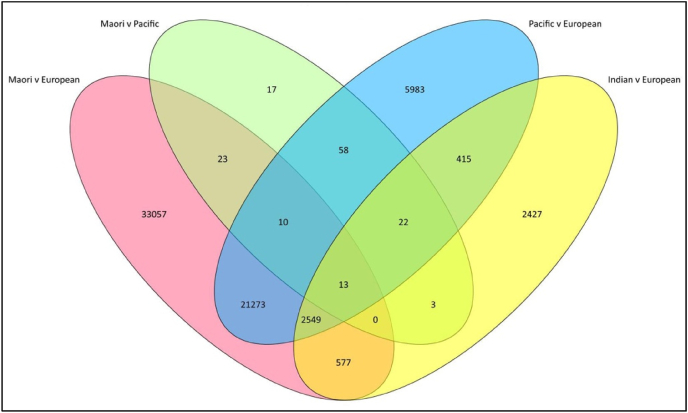
**Venn diagram summarising the number of DNAm sites that were significantly different between specific ethnicities in the MENZACS cohort and the relationship between comparisons.** An EWAS was conducted using ethnicity as comparator. The threshold for a site to be considered significantly different between populations groups was (false discovery rate corrected) P-value <0.0001. As expected, due to known similarities in ancestral population structure, there was a strong overlap between Māori and Pacific in terms of DNAm sites that had significantly higher or lower levels of DNAm compared with Europeans.

Given that population genetic variation was identified as a potential risk by our SWOT analysis, we next agreed to examine whether genetic effects were a possible confounder of specific DNAm markers in this multi-ethnic Aotearoa New Zealand cohort. To identify these potential effects, we conducted an EWAS examining DNAm differences between specific MENZACS populations. Of note, the only EPIC v1 CpG sites excluded from this analysis were those previously identified as cross-reactive and non-specific (Pidsley et al., 2016c), with known polymorphic sites being retained. From this we observed ethnicity-associated differential DNAm effects at approximately 65,000 CpG sites (Fig. 1), representing 7.6 % of the total (approximately 850,000) sites on the Illumina EPIC array. In contrast, only 146 CpG sites were differentially methylated between Māori and Pacific participants, an observation that is consistent with the known similarities in ancestral population structure between these two groups.

**Fig. 2:**
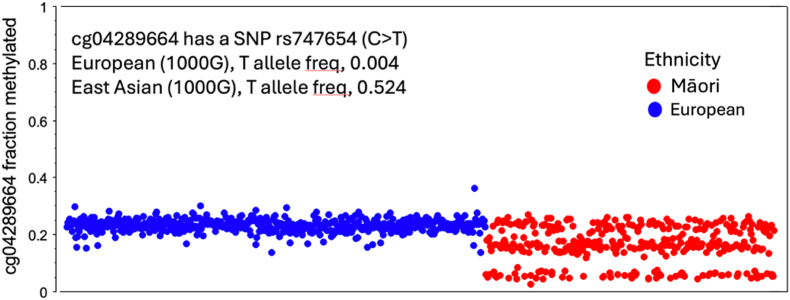
**A population-specific genetic effect on DNAm that is not present in Europeans at DNAm (CpG) site cg04289664**. In this plot each dot represents the cg04289664 DNAm β-value for an individual MENZACS study participant. This CpG site contains a single nucleotide polymorphism in non-European populations (rs747654 C > T), which manifests as the trimodal distribution observed in Māori, reflecting the different rs747654 genotypes of these individuals. If the discovery EWAS was conducted exclusively within European participants this DNAm site would not be flagged as polymorphic, and therefore subject to genetic variant confounding, due to its apparent unimodal distribution.

**Fig. 3:**
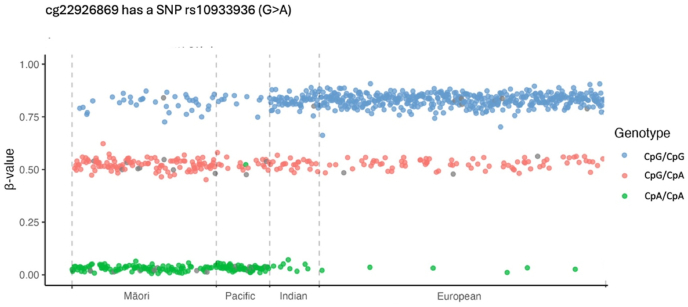
**A population-specific genetic effect on DNAm resulting from differing allele frequencies at DNAm (CpG) site cg22926869**. This plot shows the DNAm β-values for the DNAm (CpG) site cg22926869. This methylation site is known to contain a single nucleotide polymorphism (rs10933936 G > A). Consequently, individuals will carry either two, one or no intact CpG sites depending on their rs10933936 genotype. Of note, even when such a genetic effect is readily apparent the effect on DNAm values can differ substantial between population groups depending on the polymorphism allele frequency. In this case, the allele that ablates the CpG site has a frequency of approximately 0.1 in Europeans but >0.7 in Māori and Pacific MENZACS study participants.

**Fig. 4:**
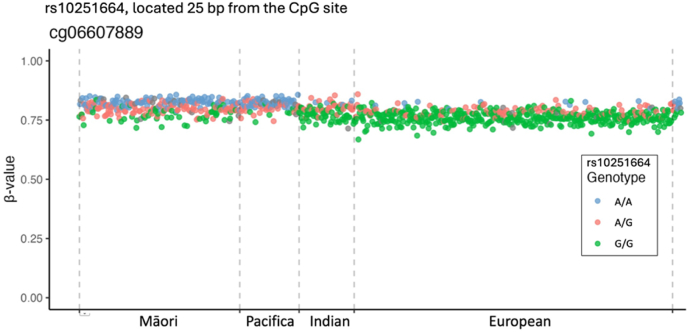
**A population-specific genetic effect on DNAm resulting from an adjacent non-CpG site genetic effect at DNAm (CpG) site cg06607889** This plot shows the DNAm β-values for the CpG site cg06607889, which does not contain any known polymorphisms. There is, however, a polymorphism (rs10251664 G > A) 25bp from the methylation site and within the detection probes DNA binding region. In this example Europeans have a G allele frequency of approximately 0.14 compared with >0.8 in Māori and Pacific participants. Note that this effect does not result in a multi-modal β-value distribution, making this clearly population-related effect cryptic in nature, unless genotype is considered.

Examining the distribution of DNAm values within specific sites identified patterns consistent with genetic effects. These included patterns suggestive of genetic variation: a) only in specific populations (Fig. 2); b) across all population groups but demonstrating differing magnitudes of effect due to allele frequencies (Fig. 3), and/or c) related to genetic variation in the probe binding region neighbouring the CpG site under interrogation (Fig. 4). A strength of the analysis was that the MENZACS participants all had high quality genotyping data available (Earle et al., 2024) for cross-referencing with their DNAm values (Fig. 3, Fig. 4). This provided a high degree of confidence that population-related effects in the DNAm signal were indeed present and required careful consideration during subsequent data interpretation.

We then revisited the DNAm marker, cg05575921, in *AHRR*, to specifically determine if it displayed features suggestive of genetic variant confounding. No evidence of such effects (multi-modal distributions, distinct from self-reported smoking status) were observed for this marker (Supplementary Fig. 2).

## 5 Discussion

In this paper we highlight considerations for best practice when undertaking epigenetics studies in diverse ethnic groups and Indigenous Peoples, as exemplified by the population of Aotearoa New Zealand. Given previous literature reporting strong associations between DNAm and conditions such as type 2 diabetes and dyslipidaemia (Braun et al., 2017; Juvinao-Quintero et al., 2021) there is considerable interest regarding the potential of DNAm markers to improve chronic disease risk prediction (McCartney et al., 2018) and understanding disease mechanisms.

We conducted an exploratory Smoking Status EWAS and confirmed concordance with previous international reports. In addition, we observed strong multi-ethnic (Māori, Pacific, Asian, and Pākehā/NZ European) concordance for the top hit smoking status association, cg05575921 *AHRR*. As such, we suggest that this specific marker could have equitable multi-ethnic application. In the case of cg05575921 *AHRR* this could be utilised as a more accurate method of assessing smoking exposure beyond self-reported measures. This was an important observation as applicability of potential markers across diverse populations was highlighted as an important consideration for this study, especially given the historical tendency for genetic and epigenetic studies to underrepresent minority ethnic groups. Ensuring equitable application of potential DNAm markers across different populations is critical to avoid exacerbating existing health inequities.

Despite the strength of these findings, challenges remain in the broader implementation of DNAm markers in clinical settings. While cg05575921 *AHRR* provided valuable insights into smoking history, other DNAm markers related to cardiometabolic risk factors (e.g., diabetes, obesity, hypertension) will require further validation to determine their independent predictive value.

The potential for individual and transgenerational stigmatisation was identified as a key risk of this research. While DNAm markers could provide more objective measures of lifestyle exposures, such as smoking, there is a danger that this information could be misused to reinforce negative stereotypes about certain populations. For example, framing CVD risk purely in terms of individual lifestyle choices may overlook the impacts of colonisation and the broader social determinants of health that disproportionately affect Māori and Pacific peoples in Aotearoa New Zealand and Indigenous populations globally. To mitigate these risks, we adopted a range of proactive strategies. DNAm findings were framed within a broader understanding of health determinants, avoiding narratives that isolate behaviour from historical and structural context. We prioritised markers with equitable application across population groups, to reduce the risk of biased interpretation. All interpretations were undertaken with oversight from the Māori Governance Group to ensure cultural relevance and prevent deficit framing. These strategies reflect our view that DNAm is not a fixed or purely individual marker, but a socially embedded and modifiable signal. The guiding values collaboratively established were crucial in addressing these concerns, ensuring that the research remained aligned with kaupapa Māori principles of integrity, social accountability, and reciprocity.

Moreover, the issue of data sovereignty was a significant consideration throughout. Any requirement for epigenetic data to be uploaded to externally hosted servers, particularly for the calculation of some epigenetic clock scores, presents a potential conflict with the principles of Māori data sovereignty. Ongoing dialogue with the MGG was essential in ensuring that the data management practices respected the rights of Māori over their genetic material and the knowledge generated from it. In response to these concerns, generation of any analysis requiring data uploading to external servers was specifically excluded. This decision could be reconsidered in future analysis, noting the potential need for external validation or algorithm transfer, however, these discussions would need to be overseen by the MGG with clear deference to the principles of Māori data sovereignty. These issues further underscore the need for ethical frameworks, such as Indigenous-led repositories and shared governance models, that balance scientific utility with Indigenous rights. Such considerations are part of a broader shift toward socially accountable genomic research that respects both methodological rigour and Indigenous sovereignty.

A key conclusion is that to avoid worsening existing health inequalities, the development of epigenetic (DNAm) disease risk markers must include discovery and validation phases involving all ethnicities where the markers may be applied. It is also crucial to have genetic data from the populations being studied. The MENZACS genetic dataset is particularly valuable, given the scarcity of such data in publicly available databases.

We advocate for epigenetic scientists and clinical researchers to adopt a similar value-based framework as we suggest that this approach is more likely to ensure that epigenetic research contributes to improving health outcomes for diverse populations, particularly those most impacted by health disparities.

## 6 Study limitations

This study is not without limitations. While the integration of ethical, cultural, and technical considerations strengthens its relevance, the sample size particularly for specific Pacific and Asian populations limits the statistical power to detect subtle DNAm associations. Although efforts were made to validate self-identified ethnicity using grandparental data, limitations inherent in NHI-recorded ethnicity may still influence subgroup classification. Finally, while our values-based framework is grounded in kaupapa Māori and aligns with Te Mana Raraunga and CARE principles, further work is required to formally evaluate its transferability to other contexts and Indigenous communities.

Overall, this research highlights the importance of applying a culturally informed, ethically grounded framework when conducting epigenetic research, especially within indigenous and multi-ethnic cohorts. This need for equity-aware interpretation aligns with recent work which demonstrated how socioeconomic and racial context shapes the interpretation of epigenetic aging biomarkers, reinforcing the importance of embedding social determinants into analytic frameworks (Maunakea et al., 2024). By adopting this values-based approach, the study not only contributes to the scientific understanding of DNAm markers in CVD but also advocates for more inclusive, equitable, and socially responsible research practices in future studies.

## CRediT authorship contribution statement

**Anna Rolleston:** Writing – original draft, Methodology, Investigation, Funding acquisition, Data curation, Conceptualization. **Gregory T. Jones:** Writing – original draft, Funding acquisition, Formal analysis, Data curation, Conceptualization. **Nikki J. Earle:** Writing – review & editing, Formal analysis, Data curation. **Sam Gibbs:** Writing – review & editing, Formal analysis, Data curation. **Anna Pilbrow:** Project administration, Formal analysis, Data curation. **Allamanda Faatoese:** Writing – review & editing, Methodology, Funding acquisition, Formal analysis, Data curation. **Katrina K. Poppe:** Writing – review & editing, Methodology, Funding acquisition, Formal analysis, Data curation. **Kimiora Henare:** Writing – review & editing, Methodology, Funding acquisition, Formal analysis, Data curation. **Vicky A. Cameron:** Writing – review & editing, Methodology, Funding acquisition, Formal analysis, Data curation. **Donia Macartney-Coxson:** Writing – review & editing, Methodology, Funding acquisition, Formal analysis, Data curation. **Malcolm E. Legget:** Methodology, Funding acquisition, Formal analysis, Data curation. **Robert N. Doughty:** Writing – review & editing, Methodology, Investigation, Funding acquisition, Formal analysis.

## Ethics statement

This study was approved by the New Zealand Health and Disability Ethics Committee (HDEC), reference number **2025 PR 4372**. All participants provided written informed consent, and all research activities were conducted in accordance with applicable ethical guidelines and institutional protocols.

## Funding

MENZACS is supported by grants from the 10.13039/100002129Heart Foundation of New Zealand (Heart Health Research Trust grant 1957), Healthier Lives National Science Challenge (Ministry of Business Innovation and Employment Reference UOOX1410 and UOOX1902), Green Lane Research and Educational Fund (17/26/4130), Freemasons Foundation and the 10.13039/501100001537University of Auckland.

## Declaration of competing interest

The authors declare the following financial interests/personal relationships which may be considered as potential competing interests:Greg Jones reports financial support was provided by Healthier Lives National Science Challenge. If there are other authors, they declare that they have no known competing financial interests or personal relationships that could have appeared to influence the work reported in this paper.

## Data Availability

The authors do not have permission to share data.
