# Supplementary material for: Considerations for study design and analysis for ethically and culturally safe DNA methylation research in Aotearoa New Zealand
Source: SSM Popul Health. 2025 Dec 4;33:101889. doi: 10.1016/j.ssmph.2025.101889 (PMC12723301; doi:10.1016/j.ssmph.2025.101889)
Supplement: Multimedia component 1 [file mmc1.docx]

**Methods**

**Study design and participants**

We conducted an epigenome-wide association study (EWAS) to identify DNA methylation markers associated with self-reported tobacco smoking status (current/ex-smoker/never) across multiple ethnicities.

A subset of 979 participants from the Multi-Ethnic New Zealand Study of Acute Coronary Syndrome (MENZACS) were available for this analysis (Table 1 below). All participants provided written informed consent. This study was approved by the Health and Disability Ethics Committee (New Zealand) and conducted in accordance with the Declaration of Helsinki

Whole blood genomic DNA, self-reported smoking phenotype data (current/ex-smoker/never), and demographic covariates (age, sex, self-identified ethnicity: Māori, Pacific, Indian or European) were available.

The EWAS examined in this study were conducted using previously described methods on the Illumina Infinium MethylationEPIC BeadChips (version1).^1, 2^ In brief, peripheral whole blood was collected in EDTA tubes and used for DNA extraction. Five hundred nanograms of genomic DNA per sample were bisulfite-converted and hybridised to Illumina EPIC arrays following manufacturer recommendations. Raw idat files were processed using the Illumina GenomeStudio software, and the Bioconductor packages ChAMP^3^ and minifi (v1.46)^4^. GenomeStudio background correction and normalisation were applied. Any sample flagged as having poor intensity in either the red or green fluorescence channel, probes reported to be cross-reactive and non-specific probes^5^ were excluded from the analyses. However, probes for known polymorphic CpG sites ^5^ were retained as these were potentially relevant to the subsequent multi-ethnic comparisons

White blood cell composition (BCC) proportions were estimated from array methylation data using the Houseman extended reference-based method.^6, 7^ These estimates, along with chronological age, biological sex, array slide and array position, were used as covariates in the EWAS analysis to minimise confounding by blood cell heterogeneity.

Adjustment for multiple comparisons was made using a false discovery rate (Benjamini–Hochberg) approach and were reported as q-values (significant <0.05).

Methylation β-values were transformed to M-values for subsequent differential statistical analysis. However, unadjusted β-values were used in graphical analysis and biological interpretation.^8^

Each EWAS participant also had genotype data available for analysis. This was generated using the Illumina Infinium Global Screening Array (v3.0) (Illumina Inc. San Diego, CA). Over 650,000 SNPs are present on the array with high genomic coverage of diverse populations from the 1000Genomes project phase III. Imputation was carried out using the Michigan Imputation Server (<https://imputationserver.sph.umich.edu/>). Over 30M markers had an imputation quality (r^2^) of >0.3 (suggesting acceptable quality).

DNAm CpG sites with β -values suggestive of SNP effects were investigated using external annotation information (Illumina methylEPIC v1.0 B5 Manifest File, dbSNP, and the UCSC Genome Browser). Genotype data for SNPs potentially influencing probe binding or CpG sites were requested, following consultation and pre-approval by both the MENZACS Governance Group and the MENZACS Māori Governance Group.

References:

1. Jones GT, Marsman J, Bhat B, Phillips VL, Chatterjee A, Rodger EJ, Williams MJA, van Rij AM and McCormick SPA. DNA methylation profiling identifies a high effect genetic variant for lipoprotein(a) levels. *Epigenetics*. 2020;15:949-958.

2. Cameron VA, Jones GT, Horwood LJ, Pilbrow AP, Martin J, Frampton C, Ip WT, Troughton RW, Greer C, Yang J, Epton MJ, Harris SL and Darlow BA. DNA methylation patterns at birth predict health outcomes in young adults born very low birthweight. *Clinical Epigenetics*. 2023;15:47.

3. Tian Y, Morris TJ, Webster AP, Yang Z, Beck S, Feber A and Teschendorff AE. ChAMP: updated methylation analysis pipeline for Illumina BeadChips. *Bioinformatics*. 2017;33:3982-3984.

4. Aryee MJ, Jaffe AE, Corrada-Bravo H, Ladd-Acosta C, Feinberg AP, Hansen KD and Irizarry RA. Minfi: a flexible and comprehensive Bioconductor package for the analysis of Infinium DNA methylation microarrays. *Bioinformatics*. 2014;30:1363-9.

5. Pidsley R, Zotenko E, Peters TJ, Lawrence MG, Risbridger GP, Molloy P, Van Djik S, Muhlhausler B, Stirzaker C and Clark SJ. Critical evaluation of the Illumina MethylationEPIC BeadChip microarray for whole-genome DNA methylation profiling. *Genome Biol*. 2016;17:208.

6. Houseman EA, Accomando WP, Koestler DC, Christensen BC, Marsit CJ, Nelson HH, Wiencke JK and Kelsey KT. DNA methylation arrays as surrogate measures of cell mixture distribution. *BMC bioinformatics*. 2012;13:86.

7. Jaffe AE and Irizarry RA. Accounting for cellular heterogeneity is critical in epigenome-wide association studies. *Genome Biol*. 2014;15:R31.

8. Du P, Zhang X, Huang CC, Jafari N, Kibbe WA, Hou L and Lin SM. Comparison of Beta-value and M-value methods for quantifying methylation levels by microarray analysis. *BMC Bioinformatics*. 2010;11:587.

**Suppl Table 1. Demographics and DNAm analysis within different self-reported ethnic groups.**

|  | **All participants** | **Māori** | **Pacific** | **Indian** | **European** |
| --- | --- | --- | --- | --- | --- |
| **Participants, n** | 979 | 265 | 98 | 91 | 525 |
| **Never** | 377 (38.5) | 54 (20.4) | 33 (33.7) | 62 (68.1) | 228 (43.4) |
| **Ex-smoker** | 286 (29.2) | 92 (34.7) | 33 (33.7) | 20 (22.0) | 141 (26.9) |
| **Current** | 316 (32.3) | 119 (44.9) | 32(32.7) | 9 (9.9) | 156 (29.7) |
| **Age** | 56.3 ± 10.3 | 56.9 ± 10.1 | 52.1 ± 10.6 | 53.4 ± 11.3 | 57.2 ± 10.0 |
| **Sex, male (%)** | 707 (72.2) | 171 (64.5) | 80 (81.6) | 81 (89.0) | 375(71.4) |
|  |  |  |  |  |  |
| **cg05575921** | 0.814  (0.635-0.868) | 0.709  (0.595-0.829) | 0.815  (0.671-0.856) | 0.849  (0.788-0.873) | 0.837  (0.650-0.877) |
| **cg05575921**  **Current vs Never ROC*** | 0.978  (0.964 - 0.987)  94.34, 95.00  ≤0.785 | 0.939  (0.892 - 0.970)  98.31, 86.79  ≤0.796 | 0.954  (0.870 - 0.991)  87.50, 96.87  ≤0.774 | 0.987  (0.926 - 1.000)  100.00, 93.44  ≤0.781 | 0.985  (0.968 - 0.995)  94.87, 96.04  ≤0.806 |

* AUC (95% confidence interval), Sensitivity, Specificity, cg05575921 optimal association criterion for current smoker status.

**Supplementary Figure 1**. Boxplot comparison of cg05575921 AHRR DNAm values within self-reported smoking status groups.

Māori Pacific Indian European

**Supplementary Figure 2**. Scatterplot comparison of cg05575921 AHRR DNAm values (ordered by ascending DNAm values). Self reported smoking status appears concordant with DNAm values in all population groups and there is no indication of a multi-modal distribution suggestive of a polymorphic CpG site.
